# Predisposing and enabling factors associated with public denture service utilization among older Thai people: a cross-sectional population-based study

**DOI:** 10.1186/s12903-019-0923-1

**Published:** 2019-10-15

**Authors:** Nareudee Limpuangthip, Supaboon Purnaveja, Tewarit Somkotra

**Affiliations:** 1 Department of Prosthodontics, Faculty of Dentistry, Chulalongkorn University, 34 Henri-Dunant Rd, Patumwan, Bangkok, 10330 Thailand; 2 Department of Community Dentistry, Faculty of Dentistry, Chulalongkorn University, 34 Henri-Dunant Rd, Patumwan, Bangkok, 10330 Thailand

**Keywords:** Andersen model, Denture service, Older people, Oral health, Tooth loss, Socioeconomic inequalities

## Abstract

**Backgrounds:**

Tooth loss is one of the major oral health problems among older Thai people. However, there is the existence of socioeconomic-related inequalities in dental service utilization, especially denture service. The aim of this study was to assess the determinants associated with inequalities in denture service utilization among older Thai people using the Andersen Behavioural model.

**Method:**

This cross-sectional study involved secondary data analysis of the 2014 survey of older Thai people (*N* = 38,695). The dependent variable was a public denture service utilization over the past 5 years. Determinants were classified as predisposing and enabling factors. Predisposing variables included age, sex, education, economic condition and dependency status. Enabling variables included working status, health behaviours, health care utilization experience and social/community support. Data were analysed by using bivariate and multivariable analyses at α = 0.05. For bivariate analysis, chi-square test was used to determine the association between dependent and each independent variable. Then, all variables were incorporated into a multivariable binary logistic regression.

**Results:**

The odds of denture service utilization were significantly higher for individuals who were older, female, and had a higher educational level and health-promoting behaviors. A positive dose-response relationship was demonstrated between denture service utilization and increased quartile of household assets.

**Conclusions:**

Predisposing variables had a greater impact than enabling variables in denture service utilization among older Thai people. Despite free public denture service, socioeconomic-related inequalities persist. The government needs to reduce socioeconomic disparities to improve denture treatment inequality.

## Background

Tooth loss is one of the major oral health problems among older Thai people aged 60 years and above [1]. It limits chewing ability and reduces the performance of social functions. As a result, older people’s overall health and quality of life are negatively impacted [2–4]. The Thai National Oral Health survey during a past decade revealed that approximately 50 and 80% of older Thai people aged 60–74 and above 80 years have less than 20 remaining natural teeth. The data indicates dental prosthesis need among older Thai people [1, 5, 6]. This problem will continue to worsen because Thailand has been approaching a completely aged society [7, 8].

Since 2001, Thailand has implemented a policy of universal coverage in healthcare system. Therefore, a universal coverage has been achieved among older Thai people (aged 60 years and above). Older Thai people are eligible for two health insurance schemes; the Civil Servant Medical Benefit Scheme (CSMBS) and the Universal Health Coverage Scheme (UCS) [9, 10]. The CSMBS covers retired civil servant or those with a child/spouse eligible for CSMBS, and the others are eligible for the UCS (approximately 80% of older people). Both schemes provide comprehensive dental care for older people without copayment, which include acrylic-based removable partial and complete dentures. UCS beneficiaries can utilize dental care at registered primary care units (PCU), mostly community hospitals. PCU delivers dental care for older people at the district level across Thailand. Meanwhile, the higher-level facilities (general or regional hospitals) locate at the center of each province. The UCS scheme uses a referral system hierarchy, from a PCU to higher-level facilities, to deliver dental care to its beneficiaries if indicated. CSMBS can utilize dental care at any public facilities. Despite universal coverage policy, socioeconomic-related inequalities in dental utilization exist among older Thai people. The older people with higher socio-economic status utilize dental care at public facilities more than the less well-off [9].

The late King Rama IX was highly concerned of national tooth loss problem and dental prosthesis need. Therefore, in 2005, the Ministry of Public Health initiated Royal Denture Project for older Thai people (government-subsidized denture service). The government has campaigned to motivate older Thai people to utilize a free-of-charge acrylic-based removable denture service from public hospitals under CSMBS and UCS once every 5 years. The aim is to improve oral health and quality of life of older Thai people.

Recent studies have revealed the existence of socioeconomic-related inequalities in dental care utilization among older Thai people [9, 10]. There are differences between the poor and the rich in dental care utilization. The wealthier people are more likely to utilize dental care than the poor. This might be due to several barriers such as the physical impairment of older persons or limited health personnel [9, 11]. However, the factors associated with a denture service utilization among older Thai people have not been identified.

Inequality in utilizing dental care has been reported in other countries [12–15]. The underlying determinants have been described by using the Andersen Behavioural model of health care utilization, consisting of predisposing, enabling, and need factors [12, 13, 15]. Predisposing factors are existing conditions for utilizing or not utilizing dental care. Enabling factors are affected by personal/family or community influence. Need factors are conditions that laypeople or healthcare providers recognized as requiring medical treatment [15, 16]. To date, lack of study has investigated the underlying determinants for a public denture service utilization by using the Andersen Behavioural model, especially in a national population. This study aimed to assess the determinants associated with a public denture service utilization among older Thai people using the Andersen Behavioural model.

## Methods

### Study population

This study analysed secondary data collected from a national survey of older Thai people in 2014. The survey was a cross-sectional household study of people aged 50 and above, conducted by the Thai National Statistical Office (NSO). Data were collected by face-to-face interviews using a structured questionnaire that was divided into household and individual levels. Two-stage stratified sampling and survey weights were employed to ensure that calculated estimates were representative of older Thai population at regional and national levels [17]. Primary sampling units in municipal and non-municipal areas were collected in blocks and villages, respectively. In proportion with the total household numbers of corresponding blocks or villages, each household was randomly selected as a secondary sampling unit which covered 56,419 households. Systematic random samples of 15 and 10 households were then selected from each sample block and village, respectively.

Samples included 38,695 older Thai people aged 60 years and above. People aged less than 60 years were excluded because their distinct characteristics from those of the older age group could turn a variable into an effect modifier in the analysis. Of the total samples, 78.9% were self-response interviews, 5.4% were assisted interviews, and 15.4% were other family/non-family member interviews. This was because the older people had different types of limitations, such as hearing/speaking impairment, memory loss, or physical/mental illness.

The study protocol was approved by the Human Research Ethics Committee of the Faculty of Dentistry, Chulalongkorn University (study code: HREC-DCU 2017–059).

### Dependent variable

Samples were interviewed whether they utilized a denture service from government healthcare institutions (community, general, or regional hospitals) over the past 5 years. The dependent variable was defined as a public denture service utilization over the past 5 years. Denture service included a new removable partial and/or complete denture fabrication, excluding denture repair or adjustment.

### Independent variables

Independent variables consisted of predisposing and enabling variables. Predisposing variables included age groups (60–69, 70–79, and at least 80 years), sex (male, female), educational level (uneducated, primary or less, secondary or vocational, at least tertiary), economic condition (quartiles of asset index), and dependency status (no, pre−/low, moderate, and high dependency). Economic condition was determined via an asset index of household possession. The index was calculated in quartiles through a Principle Component Analysis. Household ownership of durable goods (television, radio, washing machine, microwave, oven, refrigerator, air condition, computer, car, and truck) was used for measurement.

Dependency status was assessed using four variables, including visual/hearing impairment, ability to brush teeth, ability to travel by bus/boat, and performing regular exercise. Following the Seattle Care Pathway (SCP) [18], dependency was classified into 5 levels: 1) no dependency (see and hear clearly without any aids, able to brush teeth and travel by themselves, and exercise regularly), 2) pre-dependency (see and hear clearly using visual/hearing aid, able to brush teeth and travel by themselves/with some help, no regular exercise), 3) low dependency (able to see and hear clearly with/without visual/hearing aid and travel by themselves/with some help but need some help/unable to brush teeth), 4) moderate dependency (use/do not use visual/hearing aid but unable to travel by themselves), and 5) high dependency (visual and/or hearing impairment).

Enabling variables included working status (not working, working in agricultural sectors, working in non-agricultural sectors), health-promoting behaviour (having an annual health check-up during the past year or not), health-compromising behaviour (not smoking or drinking alcohol, either or both smoking and drinking), other healthcare utilization experiences and social/community support. Healthcare utilization experiences were assessed by whether a sample utilized the following public healthcare services/subsidies or not: influenza/pneumonia vaccination, healthcare insurance for recent illness, and treatment by health personnel after a falling accident. Social/community support was assessed by whether a sample was visited by village health volunteers during the past year, received information related to older people, and participated in a community club for older adults.

### Data analysis

The survey weighting was conducted in three steps. First, design weight was computed to compensate for unequal probability of sample selection. Second, weights were adjusted for nonresponses. Third, post-adjustment calibration was performed to match the estimation of older Thai population. Factors involving in sample weights included area of residence (municipal/non-municipal area) from each region of Thailand (Bangkok, Central, North, North-East, and South), and household numbers in each block and village in the area. Sample weights were applied in both descriptive and inferential analyses. Descriptive analysis was calculated to determine the percentage of samples who utilized public denture service according to independent variables. Bivariate analysis was carried out to determine the associations between denture service utilization and each independent variable by using chi-square test. Then, all variables were incorporated simultaneously into a multivariable binary logistic regression model. All data were analysed using STATA version 13.0 at α = 0.05.

## Results

### Sample characteristics

For predisposing variables, 55.7% of the participants were in a younger age group, while the oldest age group included approximately 15% of the sample (Table 1). Most of them had a primary educational level, and nearly 40% were currently working. The number of older people in the 1st quartile of household assets was nearly two times greater than the other quartiles. The majority were pre−/low-dependent older people, while approximately 15% had moderate-to-high dependency.

**Table 1:** Distribution of samples according to public denture service utilization over the past five years

Variables	Overall distribution (%)	Utilizing public denture service (%)	Not utilizing public denture service (%)
*Overall (N = 38,695)*	*100.0*	*22.8*	*77.2*
Predisposing variables			
Age (years):*			
60–69	55.7	47.1	58.2
70–79	30.7	36.2	29.1
80+	13.6	16.7	12.7
Sex:*			
Male	44.3	40.1	45.5
Female	55.7	59.0	54.5
Educational level:*			
Uneducated	11.1	10.4	11.4
Primary or less	76.2	71.2	77.7
Secondary or Vocational	8.1	11.4	7.0
At least tertiary	4.6	7.0	3.9
Asset index of household possession:*			
1st quartile	36.8	27.1	39.8
2nd quartile	20.9	17.7	21.9
3rd quartile	20.6	22.0	20.1
4th quartile	21.7	33.2	18.2
Dependency level (Seattle Care Pathway):*			
No dependency	20.4	16.2	21.6
Pre−/Low dependency	64.7	69.8	65.0
Moderate dependency	8.5	8.9	8.3
High dependency	6.4	5.1	5.1
Enabling variables			
Working status:*			
Economically inactive	58.8	56.9	65.3
Economically active:			
Working in agricultural and related sectors	22.3	24.5	14.8
Working in non-agricultural sectors	18.9	18.6	19.9
Health behaviors:			
Health-promoting behavior (Annual health check-up):*			
No	47.2	42.4	48.7
Yes	52.8	57.6	51.3
Health-compromising behavior:*			
Alcohol drinking and Smoking:			
Neither	79.2	84.6	77.6
Either/Both	20.8	15.4	22.4
Healthcare utilization experience:			
Public service utilization (vaccination):*			
No	78.5	76.1	79.2
Yes	21.5	23.9	20.8
Utilization of healthcare service for recent illness:*			
No illness	74.6	71.6	75.5
Illness: Insurance use	22.8	25.2	22.2
Non-insurance use due to minor illness	0.8	0.6	0.8
Non-insurance use due to other reasons	1.8	2.6	1.5
Treatment by health personnel for recent falling accident:*			
No falling	88.6	86.9	89.1
Falling: Receive treatment	2.7	3.2	2.6
No treatment due to minor accident	6.1	7.0	5.8
No treatment but take care themselves	2.6	2.9	2.5
Social/community support:			
Visited by village health volunteer:*			
No	44.1	49.4	42.6
Yes	55.9	50.6	57.4
Information awareness:			
No	11.6	12.1	11.4
Yes (at least one source)	88.4	87.9	88.6
Participating the community club for older adults:*			
No	65.0	67.5	64.2
Yes	35.0	32.5	35.8

*Statistical significance between group comparisons after chi-square test (*p* < 0.001)

For enabling variables, nearly half of the participants regularly had an annual health check-up, while one-fifth of them were current smokers and/or alcohol drinkers. In the event of an illness, 90% of the participants utilized government healthcare insurance. More than half of the participants were visited by health volunteers and commonly received information designed for older people (88.4%). Approximately one-third of them participated in community clubs.

### Characteristics of denture service utilization

The overall rate of denture service utilization was 22.8% (Table 1). For predisposing variables, the percentage of denture service utilization in the middle−/oldest-old age and older Thai with at least secondary education were about 1.5 times higher than their counterparts. Percentage of denture service utilization increased with higher quartile of household asset.

For enabling variables, participants who were working in agricultural sector had the lowest prevalence in denture service utilization (15.2%) compared with other working groups. Percentage of denture service utilization in participants with health-enhancing behaviours was up to 39.9%, while those with health-compromising behaviours was calculated at only 16.9%. Older Thai people who utilized healthcare insurance for recent illness or received treatment after falling accident utilized denture service more frequently than the average. Percent denture service utilization in participants who regularly visited by village health volunteer, participated community club, or had information awareness was lower than the average.

The unadjusted and adjusted odds ratio for denture service utilization of the independent variables showed similar trends of association (Table 2). The odds of denture service utilization were significantly greater in groups of older age, females, and those with higher educational levels. A positive dose-response relationship was demonstrated between denture service utilization and increased quartile of household assets. The highest odds of denture service utilization were shown in the pre−/low-dependent samples, and the lowest were in the high dependent subgroup.

**Table 2:** Adjusted odds ratio with 95% CI of denture service utilization over the past 5 years (*N* = 38,695)

Variables	Denture service utilization
	aOR (95% CI)
Predisposing variables	
Age (years):	
60–69	1 (Ref)
70–79	1.71 (1.61, 1.81)***
80+	1.96 (1.80, 2.13)***
Sex:	
Male	1 (Ref)
Female	1.26 (1.19, 1.33)***
Educational level:	
Uneducated	1 (Ref)
Primary or less	1.13 (1.04, 1.23)**
Secondary or Vocational	1.44 (1.28, 1.63)***
At least tertiary	1.26 (1.08, 1.48)**
Asset index of household possession:	
1st quartile	1 (Ref)
2nd quartile	1.21 (1.13, 1.30)***
3rd quartile	1.61 (1.50, 1.73)***
4th quartile	2.24 (2.08, 2.40)***
Dependency level (Seattle Care Pathway):	
No dependency	1 (Ref)
Pre−/Low dependency	1.26 (1.18, 1.34)***
Moderate dependency	0.98 (0.87, 1.27)
High dependency	0.72 (0.64, 0.82)***
Enabling variables	
Working status:	
Economically inactive	1 (Ref)
Economically active:	
Working in agricultural and related sectors	0.70 (0.61, 0.79)***
Working in non-agricultural sectors	0.99 (0.88, 1.10)
Health behaviors:	
Health-promoting behavior (Annual health check-up):	
No	1 (Ref)
Yes	1.44 (1.23, 1.72)***
Health-compromising behavior:	
Alcohol drinking and Smoking:	
Neither	1 (Ref)
Either/Both	0.84 (0.79, 0.91)***
Healthcare utilization experience:	
Public service utilization (vaccination):	
No	1 (Ref)
Yes	1.17 (1.10, 1.24)***
Utilization of healthcare service for recent illness:	
No illness	1 (Ref)
Illness: Insurance use	1.16 (1.09, 1.23)***
Non-insurance use due to minor illness	0.80 (0.59, 1.08)
Non-insurance use due to other reasons	1.49 (1.25, 1.77)***
Treatment by health personnel for recent falling accident:	
No falling	1 (Ref)
Falling: Receive treatment	1.13 (0.98, 1.31)
No treatment due to minor accident	1.23 (1.11, 1.36)***
No treatment but take care themselves	1.19 (1.02, 1.38)*
Social/community support:	
Visited by village health volunteer:	
No	1 (Ref)
Yes	0.78 (0.74, 0.83)***
Information awareness:	
No	1 (Ref)
Yes (at least one source)	0.99 (0.92, 1.07)
Participating the community club for older adults:	
No	1 (Ref)
Yes	0.95 (0.89, 0.99)*

*aOR* adjusted odds ratio, *95% CI* 95% confidence interval

***p < 0.001, **p < 0.01, *p < 0.05 significant association after multivariable logistic regression

For enabling variables, participants working in an agricultural sector showed the lowest likelihood of denture service utilization compared with the other subgroups (Table 2). Higher odds were shown in participants who had an annual health check-up and did not smoke or drink alcohol compared with the other subgroups. Health insurance usage and treatment for falls by health personnel were insignificantly associated with denture service utilization. Respondents visited by village health volunteers or who participated in a community club for older adults were less likely to utilize denture service compared with the other subgroups.

## Discussion

The main aim of the Thai government is to increase the accessibility and utilization of oral health promotion, disease prevention and oral rehabilitation. Denture treatment offered to older people under a universal health scheme aims to reduce oral problems due to tooth loss. The program is an oral rehabilitation that is intended to improve older people’s quality of life [19].

Our study showed that all predisposing factors were associated with denture service utilization. The strongest dose-response association was shown in the quartile of the household asset index. The higher the quartile, the greater the odds of denture service utilization. Several studies have reported associations between dental service utilization and wealth, represented by the number of household assets and individual/family monthly income [13–15]. A higher asset index level was also associated with other health service utilizations, such as emergency and mental health care [20, 21]. In addition, we found that the likelihood of denture service utilization increased with higher educational level. It is possible that education is a proxy indicator for economic condition [14, 22]. Our data consistently showed that older Thai people with higher education mostly fell into the higher quartiles of household assets.

According to the 8th National Oral Health survey of Thailand, the need for removable complete dentures and partial denture treatment is mostly prevalent in older- and younger-age people, respectively [1]. Our results showed greater odds of denture service utilization in the older-age subgroup compared with their younger counterparts. This finding might be explained by the fact that younger people turn into middle- and oldest-old people. Therefore, the likelihood of denture service utilization increases in tandem with older age.

In our study, the lowest and highest odds of denture service utilization were found in high- and pre−/low dependent older people, respectively. Earlier studies determined the physical health of older people through indicators such as Activities of Daily Living (ADL), Instrumental Activities of Daily Living (IADL) and chronic conditions [12, 22, 23]. In contrast, the SCP criterion used in this study reflects both physical and cognitive impairment [18]. Our data revealed that high-dependent samples were mostly home-bound due to physical impairment, while low−/pre-dependency individuals were mostly younger people who had no physical constraints to obtaining access to health services. Because physical and cognitive impairments impede the accessibility of denture service, it is important to maintain good oral health from a pre-older age. National oral health promotion and tertiary prevention should start at the level of pre-older people.

In addition to predisposing factors, some enabling factors were associated with denture service utilization. Older people working in an agricultural sector were less likely to utilize denture service, and most of them were in the lower quartiles of the asset index. Thus, working status could be an indicative factor for individual economic status.

Older Thai people who had health concerns or health-promoting behaviours usually utilized both general and oral health services. This finding is consistent with a study of older Canadians, which found an increased frequency of dental service with a greater level of health service use [12]. The effect of health service uses due to falling or illness was insignificant as the impacts were not severe. Our findings show that health concerns and behaviours may be interchangeably used as indicative factors of denture service utilization.

Older people with health-compromising behaviour less frequently utilized denture service. It was found that smoking and drinking behaviours were related to a household asset and education. The proportion of non-smokers and non-alcohol drinkers in the highest quartile were two folds greater than those in the lower quartile. In contrast, the proportion of non-alcohol drinkers in the lowest educational level was only half of those in the higher education. It could be implied that health-compromising behaviors are not only influenced by health literacy of a person, but also their culture and environment. This finding supported the steps for achieving oral health in ageing society [24]. For instance, seeking appropriate healthcare system and giving information to policy maker, finding multi-professional and sector collaboration for sustainable system such as common risk factor approach for non-communicable diseases (NCDs), promoting people’s awareness of oral health values, and implementing outreaching oral health care by integrating to the general healthcare system.

The study found that older Thai people who received social/community support from village health volunteer visits were less likely to utilize denture service. In Thailand, health volunteers work in a primary health care in community, and therefore, are at the forefront in tackling healthcare problems [25]. From our finding, health volunteers were less likely to visit older Thai people who were in the highest quartile and higher educational levels. Therefore, older Thai people who were not visited by health volunteer were more likely to utilize denture service. This finding was in contrast to previous studies that found a positive correlation between dental service use and social network/family support [13, 22]. This is unsurprising because those studies assessed living arrangements and family support, such as living alone or receiving financial support from relatives.

Social interaction motivates older people and provides resources to seek preventive care and treatment [22]. Our study found that the likelihood of denture service utilization decreased in older people who regularly attended community clubs. Community-club participants were mostly independent, while non-participants were dependent older people with physical limitations. Thus, denture service utilization might be directly affected by individual health, rather than social/community support.

Thailand has been succeeded in the implementation of the universal health scheme, and local health organizations in terms of health promotion and tertiary prevention by providing health care services. However, socioeconomic-related inequalities in denture service utilization among older Thai people persist. Predisposing factors are key social determinants that are considered more important than enabling factors in denture service utilization. Certain predisposing factors, such as socioeconomic and physical conditions, are major obstacles. Since there is no denture service charge, the reason for not utilizing denture service could be due to low degree of oral health knowledge or dental substitution awareness. Inconvenient public transportation could be another barrier, especially older people with physical impairment. To eliminate barriers, government could take approach from downstream. Providing daily and transportation allowances and mobilizing dental clinics to rural areas could resolve short term problems. For long-term solution, the government needs to help raise income to family in agricultural sectors. Reducing income gap results in better denture service utilization.

Data from the present National Survey ensure the sample representativeness and generalizability of the findings to older Thai people. The findings would be beneficial for other populations, particularly in developing counties. However, there were limitations in this study. A causal relationship between denture service utilization and underlying determinants cannot be concluded due to a cross-sectional study design. Need factors such as oral health status which might affect denture service utilization [14, 26], were not included in the analyses, because data were not available from the national survey of older Thai people. According to the national oral health surveys, however, older Thai people aged 60–74 and above 80 years with at least 4 remaining posterior occluding pairs were averagely 10 and 40%, respectively [1, 5, 6]. Thus, the needs for denture in older Thai people are in the range from 60 to 90%, and they are no differences among regions [1]. Needs were determined through other proxy indicators such as age, dependency level, and healthcare utilization experience. Under the universal coverage policy, a principle of horizontal equity for healthcare utilization is equal treatment for equal need. Thus, this study paid more attentions on predisposing and enabling than need factors.

## Conclusions

Despite the availability of free public denture service, socioeconomic-related inequalities persist among older Thai people. Predisposing variables, especially economic condition and education, had greater impacts than enabling variables on denture service utilization. In addition to a provision of universal health coverage, the government needs to reduce socioeconomic disparities to lessen denture service inequality in older people.

## Data Availability

The datasets generated and/or analysed during the current study are not publicly available due to having no signing agreement between the journal and the Thai National Statistical Office, but are available from the corresponding author on reasonable request.

## Abbreviations

aOR: Adjusted odds ratio
CI: Confidence interval
CSMBS: Civil Servant Medical Benefit Scheme
NSO: National Statistical Office
SCP: Seattle Care Pathway
SSS: Social Security Scheme
UCS: Universal Health Coverage Scheme
